# Correlation between Antioxidant Activities and Phenolic Contents of Radix Angelicae Sinensis (Danggui)

**DOI:** 10.3390/molecules14125349

**Published:** 2009-12-21

**Authors:** Xican Li, Xiaoting Wu, Ling Huang

**Affiliations:** School of Chinese Herbal Medicine, Guangzhou University of Chinese Medicine 510405, Guangzhou, China; E-Mails: idj811@hotmail.com (X.W.); hlkok26@foxmail.com (L.H.)

**Keywords:** Radix Angelicae Sinensis, antioxidant, phenolic, correlation

## Abstract

Radix Angelicae Sinensisis (RAS) is one of the most popular traditional Chinese herbal medicines. In the present study, six RAS extracts (*i.e.*, phenolic extract PE, petroleum ether extract PEE, ethyl acetate extract EAE, absolute ethanol extract AEE, 95% ethanol extract 95 EE, and water extract WE) were prepared and their antioxidant activities measured by DPPH (1,1-diphenyl-2-picrylhydrazyl radical), ABTS [2,2′-azino-bis(3- ethylbenzothiazoline-6-sulfonic acid diammonium salt)], Reducing power, •O^2–^ and lipid peroxidation assays. In general, PE, PEE and EAE had relatively high antioxidant activity, followed by AEE with moderate activity, as compared with 95 EE and WE that had low activity. Their phenolic contents (including total phenolic, ferulic acid, caffeic acid, same as below) were then determined by HPLC or spectrophotometry. The sequence of phenolic contents was roughly identical with that of antioxidant activity. When the values of 1/IC_50_ of various antioxidant assays were used to evaluate the level of antioxidant of the RAS extracts, (plot between 1/IC_50_ values and phenolic contents), the correlation coefficient (R) ranged from 0.642 to 0.941, with an average value of 0.839. Significant positive correlations demonstrated that the antioxidant effects of RAS might generally be considered a result of the presence of the phenolic compounds, especially ferulic acid and caffeic acid.

## 1. Introduction

Radix Angelicae Sinensisis (RAS, named Danggui in Chinese) is the dried root of *Angelica sinensis* (Oliv.) Diels. As a Chinese herbal medicine, RAS was originally described in an ancient Traditional Chinese Medicine (TCM) classic named *Shennong’s Classic of Herbology*, in which it is classified as top grade (superior). In TCM, it is mainly used to nourish blood, regulate menstruation, promote blood circulation, relieve pain and constipation [1]. As for the menstrual cycle and treatment of menopausal symptoms caused by the hormonal changes, it can produce favorable effects, so it is also known as “female ginseng” in Europe. Even though it is good for women, it also helps to treat the heart, spleen, liver and kidneys of both men and women. RAS, together with other Chinese herbal medicines can be used for treating various diseases, such as anemia [2], hepatitis [3], ulcerative colitis [4], dermatosis [5], neuropathy [6], cancer [7], diabetes and nephroses [8]. 

It has been well established that, a number of biochemical reactions involve the generation of reactive oxygen species (ROS) in our body. Under normal conditions, the balance between the generation and diminution of ROS is controlled by the antioxidant defense system, but under certain pathological conditions, when ROS are not effectively eliminated by the antioxidant defense system, the dynamic balance between the generation and diminution of ROS is broken. Excessive ROS attack lipids, carbohydrates, proteins, DNA, and result in oxidative stress, that leads to various disorders and diseases [9]. There are several studies which reported the antioxidant properties of RAS [10,11,12]. The purpose of this work was therefore to systematically assess the antioxidant activity and its correlation with the phenolic compounds found in RSA.

## 2. Results and discussion

### 2.1. DPPH· and ABTS·^+^ scavenging activity

**Figure 1:**
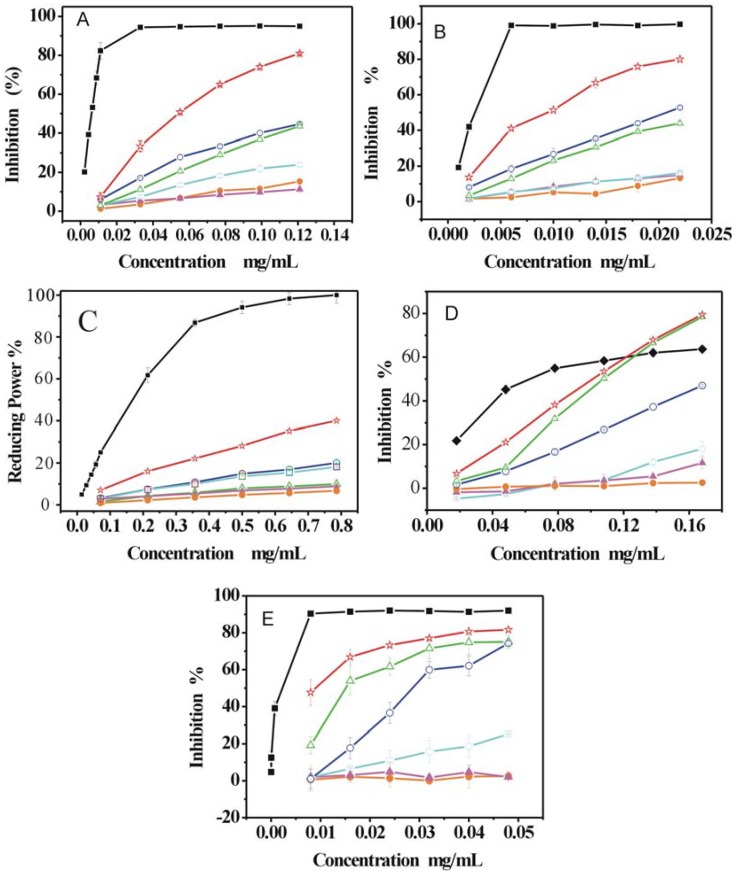
Effect of different concentration of extracts of Radix Angelicae Sinensis in free radical scavenging tests: (A) DPPH assay, (B)ABTS assay, (C) Reducing power assay,(D) Superoxide anion assay, (E) Lipid peroxidation assay.

The DPPH (1,1-Diphenyl-2-picrylhydrazyl radical) and ABTS [2,2′-azino-bis(3-ethylbenzo- thiazoline-6-sulfonic acid diammonium salt)] assays have been widely used to determine the free radical-scavenging activity of various plants and pure compounds. Both DPPH and ABTS are stable free radicals which dissolve in methanol or ethanol, and their colors show characteristic absorptions at 519 nm or 734 nm, respectively. When an antioxidant scavenges the free radicals by hydrogen donation, the colors in the DPPH and ABTS assay solutions become lighter. As presented in Figure 1A and B, both the DPPH and ABTS inhibition percentage values were dose dependent, whereby they increased in the range of the tested concentrations, for the RAS extracts and the positive control (Trolox). Both the DPPH and ABTS radicals inhibition decreased in the order Trolox > PE > EAE > PEE > AEE > WE ≈ 95 EE. The DPPH and ABTS IC_50_ values (IC_50_ value is the concentration of the sample required to inhibit 50% of radical) of the RAS extracts and Trolox were calculated and are listed in Table 1.

### 2.2. Reducing power assay

The reducing power of an extract may serve as a significant indicator of its potential antioxidant activity. It can be seen that the reducing power percentage values of all RAS extracts and the positive control (Trolox) were concentration related and increased with the increase in sample concentration in the range of the tested concentrations (Figure 1C). Their relative reducing powers were as follows: Trolox > PE > EAE > AEE > PEE > 95EE > WE. The IC_50_ values of the RAS extracts and Trolox were calculated and are listed in Table 1.

### 2.3. Superoxide anion (·O_2_ -) radical-scavenging activity

The superoxide radical was generated by the pyrogallol system at pH 8.2. Figure 1D shows the superoxide radical activity of the RAS extracts compared with the positive control (GSH). The positive control (GSH) and all RAS extracts demonstrated an ability to inhibit superoxide anion in a dose-dependent manner. At low concentrations (0.00–0.12 mg/mL), GSH had the highest inhibition percentage value, but it was exceeded by PE and PEE at high concentration (>0.12mg/mL). According to the IC_50_ values (Table 1), the superoxide radical-scavenging activity of all samples decreased in the following order: GSH > PE > PEE > EAE > AEE > 95 EE > WE.

### 2.4. Lipid peroxidation in a linoleic acid emulsion model system

Lipid peroxidation (LPO) was used because of the formation of cytotoxic products such as MDA and 4-hydroxynonenal, which can influence cell function and the course of major human diseases [13]. As shown in Figure 1E, except for AEE, 95 EE and WE, the other extracts and Trolox showed a good inhibitory activity against lipid peroxidation. Obviously, the activity of all samples occurred in the order Trolox > PE > PEE > EAE > AEE > 95 EE > WE. The LPO IC_50_ values were calculated and are listed in Table 1.

### 2.5. Total phenolic content

The total phenolic contents of RAS extracts were determined and are presented in Table 2. The phenolic contents were calculated using pyrogallol. Analysis of the phenolic content in all of the extracts using the Folin-Ciocalteu method revealed that PE contained the maximum phenolic content (30.20 ± 0.18 mg/g) in terms of pyragallol equivalents (PYR.), followed by EAE (29.57 ± 0.37 mg/g), PEE (28.83 ± 0.64 mg/g), AEE (15.65 ± 0.68 mg/g), 95EE (5.310.69 mg/g), and WE(2.26 ± 0.55 mg/g).

### 2.6. Determination of ferulic acid and caffeic acid by HPLC

**Figure 2:**
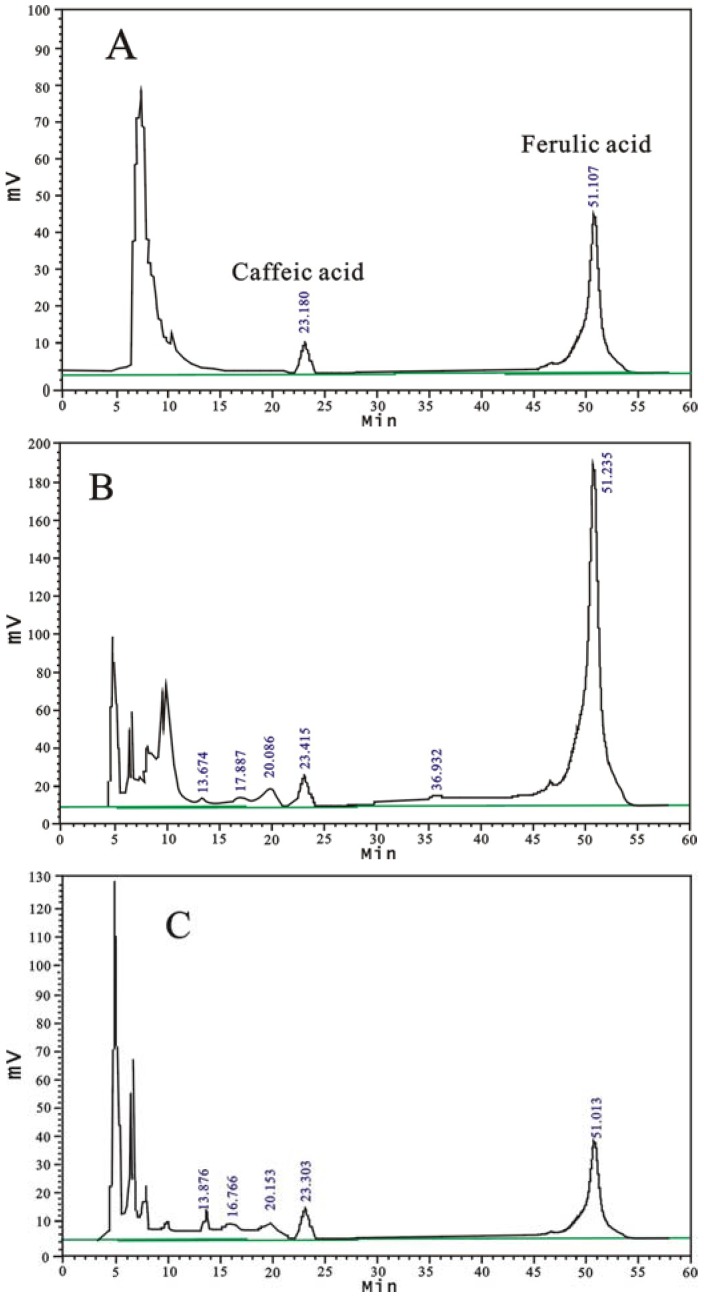
HPLC chromatograms of: (A) caffeic acid and ferulic acid (the standards), (B) ethyl acetate extract (EAE) and (C) water extract (WE).

As the common active phenolic acid compounds, ferulic acid and caffeic acid had been earlier found in RAS and their antioxidant effects have also been reported in previous studies [14,15]. In the present study, the RAS extracts were assayed by chromatographic analysis, using the HPLC-UV, to determine the contents of ferulic acid and caffeic acid. Typical chromatograms of the standard compounds and EAE, WE are shown in Figure 2. The calibration curves of ferulic acid and caffeic acid also showed good linearity (R = 0.9997 and 0.9998, respectively) (Table 3). As shown in Table 2, the results indicated that the ferulic acid content of PE (510.00 ± 7.00 μg/g) was highest among the RAS extracts, followed by EAE (41.00 ± 1.70 μg/g) as compared with 95 EE (8.80 ± 0.74 μg/g)*,* PEE (6.40 ± 0.14 μg/g), AEE (5.20 ± 0.18 μg/g) and WE (0.81±0.013 μg/g), respectively. Moreover, PE also had the highest caffeic acid content (160.00 ± 2.30 μg/g) on comparing it with PEE (8.00 ± 0.20 μg/g), AEE (5.90 ± 0.31 μg/g), EAE (4.60 ± 0.12 μg/g), WE (1.00 ± 0.11 μg/g) and 95 EE, which showed complete absence of caffeic acid.

## 3. Experimental

### 3.1. Plants material

Radix Angelicae Sinensis was purchased from the Yanghe Pharmacy of Guangzhou University of Chinese Medicine (Guangzhou, China), and authenticated by Professor Shuhui Tan. A voucher specimen was deposited in our laboratory.

### 3.2. Chemicals

1,1-Diphenyl-2-picrylhydrazyl radical (DPPH·), pyrogallol, linoleic acid, Trolox [(±)-6- hydroxyl-2,5,7,8-tetramethlychromane-2-carboxylic acid], Folin & Ciocalteu’s phenol reagent were purchased from Sigma Co. 2,2′-Azino-bis(3-ethylbenzothiazoline-6-sulfonic acid diammonium salt) (ABTS) and glutathione (GSH) were obtained from Amresco Co.; ferulic acid (NO.110773 -200611) and caffeic acid (NO.110885-200102) were obtained from NICPBP (National Institute for the Control of Pharmaceutical and Biological Products, China); acetonitrile and water were of HPLC grade. All other reagents were of analytical grade.

### 3.3. Preparation of different extracts of Radix Angelicae Sinensis

**Figure 3:**
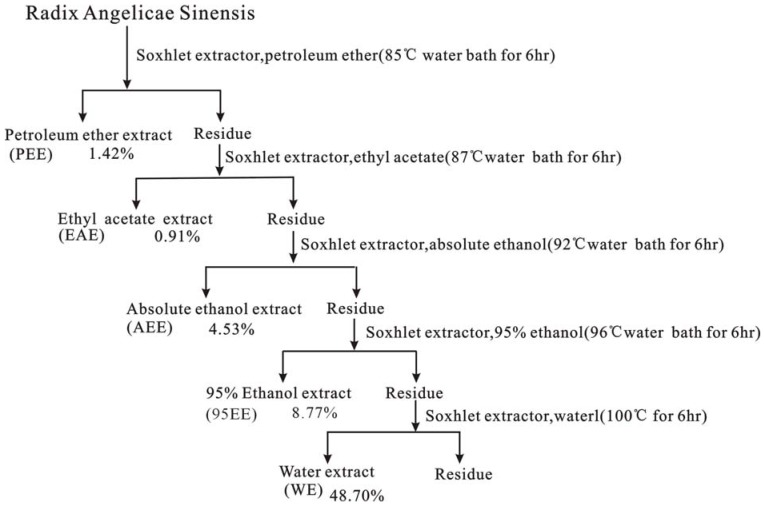
Extraction of Radix Angelicae Sinensis powder by increasing order solvent polarity.

**Figure 4:**
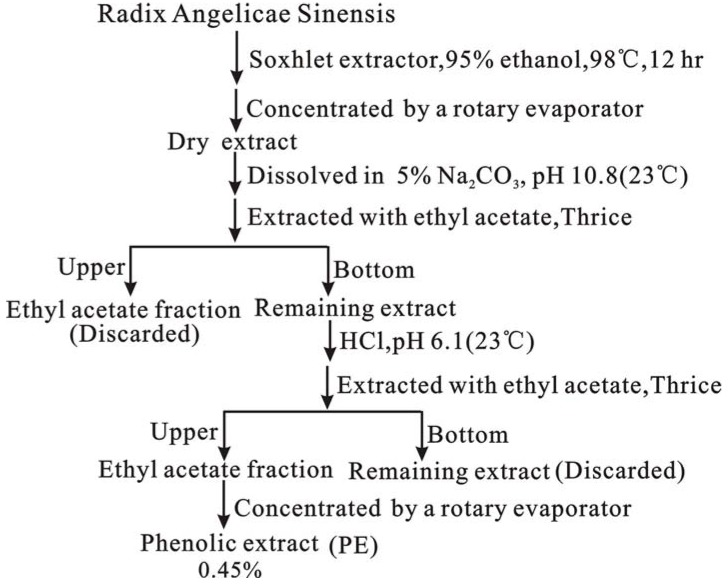
The preparation of phenolic extract (PE) of Radix Angelicae Sinensis using a pH adjustment method.

Radix Angelicae Sinensis (RAS) was ground to a coarse powder. Then the powdered sample was first extracted sequentially in the order of petroleum ether (b.p.60–90 ºC), ethyl acetate, absolute ethanol, 95% ethanol, and water to obtain the petroleum ether extract (PEE), ethyl acetate extract (EAE), absolute ethanol extract (AEE), 95% ethanol extract (95 EE), and water extract (WE), respectively (Figure 3). In addition, a phenolic extract (PE) of Radix Angelicae Sinensis was prepared using the pH adjustment method (Figure 4).

### 3.4. DPPH radical-scavenging activity

DPPH radical-scavenging activity was determined as described [16,17]. Briefly, DPPH·solution (1 mL, 0.1 mmol/L) was mixed with various concentrations of samples (0.5 mL) dissolved in suitable solvents (absolute or 95% ethanol). The mixture was kept at room temperature for 30 min, and then the absorbance at 519 nm was measured on a spectrophotometer (UNICO 2100, Shanghai, China) using 95% ethanol as the blank. GSH was used as the positive control, and the percentage DPPH· inhibition of the test samples was calculated as:

Inhibition % = (1 – *A*/*A*_0_) × 100

where *A*_0_ is the absorbance at 519 nm of DPPH without sample, and *A* is the absorbance at 519 nm of the reaction mixture containing DPPH and sample.

### 3.5. ABTS·^+^ radical cation scavenging activity

The scavenging activity of ABTS·^+^ was measured as described [18] with some modifications [19]. The ABTS·^+^ was produced by mixing ABTS diammonium salt (0.35 mL, 7.4 mmol/L) with of potassium persulfate (0.35 mL, 2.6 mmol/L). The mixture was kept in the dark at room temperature for 12 h to allow completion of radical generation, and then diluted with 95% ethanol (about 1:50) so that its absorbance at 734 nm was 0.70 ± 0.02 measured on a spectrophotometer (UNICO 2100 Spectrophotometer; Shanghai, China). To determine the scavenging activity, ABTS·^+^ reagent (1.2 mL) was mixed with sample or negative control (95% ethanol) (0.3 mL) and the absorbance at 734 nm was measured 6 min after the initial mixing, using 95% ethanol as the blank. The percentage inhibition of the samples was calculated as:

Inhibition % *=* (1 – *A*/*A*_0_) ×100

where *A*_0_ is the absorbance at 734 nm of the negative control, *A* is the absorbance at 734 nm of the mixture with sample. Trolox, with a final concentration range of 0.002–0.022mg/mL, was prepared as a standard.

### 3.6. Reducing power assay

Reducing power was determined by the method of Oyaizu [20]. Samples (*x* mL) of each extract at various concentrations were mixed with Na_2_HPO_4_/KH_2_PO_4_ buffer (3.5-*x* mL, 0.2 mol/L, pH 6.6) and K_3_Fe(CN)_6_ (2.5 mL, 1 g/100 mL). The mixture was incubated at 50 ºC for 20 min, TCA (2.5 mL, 10 g/100 mL) was added, and the mixture was centrifuged at 2,500 g for 10 min. The supernatant (2.5 mL) was recovered, mixed with distilled water (2.5 mL) and FeCl_3_ (2.5 mL, 0.1 g/100 mL) and placed immediately into the spectrophotometer (UNICO 2100, Shanghai, China), and the timer was started. The absorbance at 700 nm was measured at 90 s. Samples were analyzed in groups of three, and when the analysis of one group has finished, the next group of three samples were mixed with FeCl_3_ to avoid oxidization by air. GSH was used as the positive control, and an increased absorbance reading indicated increased reducing power. The percentage reduction of the sample as compared to standard, *i.e.*, GSH was calculated by using the formula (1−*A_S_* /*A_C_*) × 100. Here, *A_C_* = absorbance of standard at maximum concentration tested and *A_S_* = absorbance of sample.

### 3.7. Superoxide anion (•O_2_^–^) radical-scavenging activity for auto-oxidation of pyrogallol

The scavenging ability at pH 8.2 of all test samples was determined by the method of Marklund and Marklund [21], as described by Wang [22]. Briefly, samples were dissolved in suitable solvents (absolute or 95% ethanol) at a concentration of 2 mg/mL. The sample solution (*x* μL, where *x* = 0, 25, 50, 100, 150, 200, or 250 μL) was mixed with Tris-HCl buffer (1 475 - *x* μL, 0.05 mol/L, pH 8.2) containing EDTA (1 mmol/L) and pyrogallol (25 μL, 6 mmol/L), then shaken rapidly at room temperature. The absorbance at 325 nm of the mixture was measured (UNICO 2100, Shanghai, China) against the Tris-HCl buffer every 30 s for 5 min. The slope of the correlation of absorbance with time was calculated. The reaction mixture without added sample was used as the control. The •O_2_^–^ scavenging ability was calculated as:

(1 – Slope of sample/Slope of control) × 100 %

### 3.8. Lipid peroxidation in a linoleic acid emulsion model system

Lipid peroxidation (LPO) in a linoleic acid emulsion model system was applied to produce peroxyl radical (LOO·). The scavenging ability of the RAS extracts on LPO was assessed by a method using ammonium thiocyanate [23,24]. A linoleic acid emulsion was made by vortexing a mixture of linoleic acid (312.6 mg) with Tween20 (78.2 mg) in 30% (v/v) ethanol (20 mL). The reaction mixture consisted of linoleic acid emulsion (1.5 mL) and sample solution (0.1 mL). The total volume was adjusted to 2.0 mL with distilled water, and the reaction mixture was incubated at room temperature in an open vitreous bottle for 72 h. Samples (0.15 mL) were withdrawn from the incubated mixture and tested for lipid peroxidation. The assay consisted of sequential addition of 75% (v/v) ethanol (3.65 mL), ammonium thiocyanate (0.1 mL, 30 g/100 mL), and ferrous sulfate (0.1 mL, 0.02 mol/L in 3.6%, v/v, HCl). After the mixture was rested for 3 min, the color development was determined as the difference of absorbance at 500 nm against that of 75% ethanol in the reference cuvette (UNICO 2100, Shanghai, China). The scavenging ability on LPO was calculated as:

Inhibition % = (1 – *A*_500nm(sample)_/*A*_500nm(reference)_) × 100

### 3.9. Determination of total phenolic content (TP)

Total phenolic (TP) contents of the RAS extracts were analyzed by the Folin-Ciocalteu method [25]. In brief, samples of the extract were taken into a test-tube and made to a volume of 0.5 mL with 95% ethanol, the Folin-Ciocalteu reagent (0.5 mL, 0.25 mol/L) and the Na_2_CO_3_ reagent (1.0 mL, 150 g/L) were added, and the mixture was incubated at room temperature for 30 min. The absorbance at 760 nm of the mixture was measured (UNICO 2100 spectrophotometer, Shanghai, China), and the amount of total phenol was calculated as pyrogallol equivalents (mg PYR/g) from the calibration curve. 

### 3.10. HPLC analysis for ferulic acid and caffeic acid

HPLC analysis of the RAS extracts for ferulic acid, caffeic acid was carried out on a LC2000 system equipped with a UV detector (Techcomp Ltd., China) and a L-2200 autosampler (Hitachi, Japan). The column was a C_18_ reversed-phase column (Diamonsil 5 μm film thickness, 250 mm × 4.6 mm, Dikma Ltd., China). The separation was performed isocratically with a mobile phase consisting of 0.1% (v/v) water acetic acid and acetonitrile (83:17). The injection volume was 50 μL. The flow rate was 0.5 mL/min and detection was at 325 nm wavelength. The standard curves using ferulic acid (0.01–0.08 mg/mL) and caffeic acid (0.07−0.112 mg/mL) were constructed and quantifications were done on the basis of the standard curves of ferulic acid and caffeic acid, respectively. 

### 3.11. Statistical analysis

Data are given as the mean ± SD of three measurements. The IC_50_ values were calculated by linear regression analysis. All linear regression in this paper was analyzed by Origin 6.0 professional software.

## 4. Conclusions

Six extracts of Radix Angelicae Sinensisis dose-dependently increased the radical inhibition (or reducing power) values, suggesting that RAS possesses antioxidant activity. Therefore, RAS’s various pharmacological activities and curative effects maybe closely correlated with its antioxidant activities. However, the antioxidant activities of six RAS extracts were different within the tested concentration ranges. In general, PE, PEE and EAE had relatively high antioxidant activity, followed by AEE with moderate activity, as compared with 95 EE and WE which displayed low activity. This sequence of antioxidant activity was roughly identical to that of phenolic (including total phenolic, ferulic acid, caffeic acid) contents (Table 2). Thus, the antioxidant activity of RAS may likely be attributed to the phenolic content in each extract. This conclusion is expected, as similar observations have been reported in a large number of previous researches.

**Figure 5:**
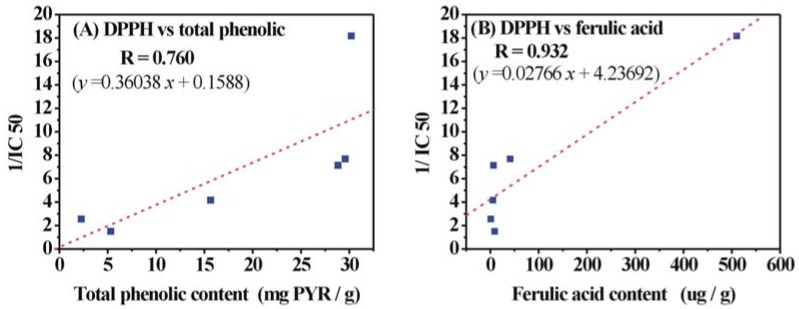
Correlation graphs for DPPH 1 /IC_50_ values and (A) total phenolic contents, (B) ferulic acid contents in the six RAS extracts.

Furthermore, quantitative analysis was also used for investigating the correlation between antioxidant activities and phenolic contents in different extracts of RAS. As the 1/IC_50_ (not IC_50_) value showed parallelism with antioxidant activity, it was therefore calculated (Table 1) and used for evaluating antioxidant activity. In the present study, twenty correlation graphs were plotted between 1/IC_50_ values (including of DPPH, ABTS, reducing power, •O_2_^–^ and LPO) and phenolic contents (including total phenolic, ferulic acid, caffeic acid and ferulic acid *plus* caffeic acid). Two typical correlation graphs (*i.e.*, DPPH *vs* total phenolic, DPPH *vs* ferulic acid) are shown in Figure 5, while Table 4 shows the correlation coefficient (R) of the various assays and phenolic contents. 

As shown in Figure 5 and Table 4, significant positive correlations (R = 0.659-0.911, the average of R was 0.772) were observed between total phenolic content and 1/IC_50_ values for DPPH, ABTS, Reducing power, •O_2_^−^ and LPO assay, indicating the significant contribution of phenolics to these antioxidant assays. As the common phenolic acid compounds, ferulic acid (the average of R was 0.860) and caffeic acid (the average of R was 0.862) exhibit higher R values between antioxidant assays than total phenol (the average of R was 0.772). Therefore, it is indicated that ferulic acid and caffeic acid exert more antioxidant capacity than other phenolic compounds in the RAS extracts. In fact, ferulic acid has been reported to possess excellent inhibitory effect on •O_2_^–^, ·OH and NO [26], and to serve an important antioxidant function in preserving physiological integrity of cells exposed to both air and impinging UV radiation [14]. Caffeic acid was also proved to be a more efficient antiradical compound than coumaric acid [17]

However, among the five antioxidant assays (*i.e.,* DPPH, ABTS, reducing power, •O_2_^−^ and LPO), the the R value of the •O_2_^−^ assay appears abnormal to some extent. Ferulic acid (R 0.642) and caffeic acid (R 0.655) exhibit lower (not higher) R values than total phenol (R 0.914). This result suggested that ferulic acid and caffeic acid exert less inhibitory effect on •O_2_^−^ than other phenolic compounds in the RAS extracts.

Nevertheless, twenty R values ranged from 0.642 to 0.941, and the average value was 0.839, so these high levels suggest that the antioxidant activity of Radix Angelicae Sinensis might me in large part the result of the contributions of the phenolic compounds that it contained, especially ferulic acid and caffeic acid.

## Figures and Tables

☆ = PE (phenolic extract); △= PEE (petroleum ether extract); ○ = EAE (ethyl acetate extract); □ = AEE (absolute ethanol extract);▲ = 95 EE (95% ethanol extract); ● = WE (water extract); ■ = Trolox (positive control ); ◆ = GSH (positive control ); Each value is expressed as mean ± standard deviation, n = 3.

**Table 1:** The values of IC_50_ and 1/IC_50_ of the extracts of Radix Angelicae Sinensis.

	***DPPH***		***ABTS***		***Reducing power***		***•O_2_^–^***		***LPO***	
	IC_50_	1/IC_50_	IC_50_	1/IC_50_	IC_50_	1/IC_50_	IC_50_	1/IC_50_	IC_50_	1/IC_50_
PE	0.055	18.18	0.008	125	1.00	1.00	0.10	10	0.0083	120.48
PEE	0.14	7.14	0.023	43.48	3.87	0.26	0.11	9.09	0.015	66.67
EAE	0.13	7.69	0.022	45.45	2.03	0.49	0.18	5.56	0.028	35.71
AEE	0.24	4.17	0.070	14.29	2.38	0.42	0.39	2.56	0.093	10.75
95 EE	0.66	1.51	0.15	6.67	5.78	0.17	0.64	1.56	0.28	3.57
WE	0.39	2.56	0.093	10.75	5.62	0.18	2.77	0.36	1.00	1.00
**Control**	0.0061*	163.93*	0.0025*	400*	0.017*	58.82*	0.061**	16.39**	0.0025*	400*

PE = phenolic extract; PEE = petroleum ether extract; EAE = ethyl acetate extract; AEE = absolute ethanol extract; 95 EE = 95% ethanol extract; WE= water extract; * Trolox; ** GSH.

**Table 2:** The phenolic contents (including total phenolic,ferulic acid, caffeic acid) of the RAS extracts.

	Phenolic* mg PYR/g	Ferulic acid* μg /g	Caffeic acid* μg /g	Ferulic acid *plus* Caffeic acid μg /g
PE	30.20 ± 0.18	510.00 ± 7.00	160.00 ± 2.30	670.00
PEE	28.83 ± 0.64	6.40 ± 0.14	8.00 ± 0.20	14.00
EAE	29.57 ± 0.37	41.00 ± 1.70	4.60 ± 0.12	46.00
AEE	15.65 ± 0.68	5.20 ± 0.18	5.90 ± 0.31	11.00
95 EE	5.31 ± 0.69	8.80 ± 0.74	0.00	8.80
WE	2.26 ± 0.55	0.81 ± 0.013	1.00 ± 0.11	1.80

* Each value is expressed as mean±standard deviation, n = 3.

**Table 3:** The calibration curve and R values of caffeic acid and ferulic acid.

Standard	Regression equation	R
Ferulic acid	*y* = 645622568*x* - 223872	0.9997
Caffeic acid	*y* = 294445216*x* -1399648	0.9998

Each value is expressed as mean±standard deviation, n = 3.

**Table 4:** The R values (correlation coefficients) between antioxidant activities (1/IC_50_) and phenolic contents.

	DPPH	ABTS	Reducing power	•O_2_^–^	LPO	**Average**
Total phenolic	0.760	0.733	0.659	0.914	0.795	**0.772**
Ferulic acid	0.933	0.941	0.929	0.642	0.856	**0.860**
Caffeic acid	0.929	0.938	0.920	0.655	0.867	**0.862**
Ferulic acid *plus* Caffeic acid	0.931	0.941	0.928	0.645	0.859	**0.861**
**Average**	**0.888**	**0.888**	**0.859**	**0.714**	**0.844**	
